# Mechanics of biomimetic free-standing lipid membranes: insights into the elasticity of complex lipid compositions†

**DOI:** 10.1039/d4ra00738g

**Published:** 2024-04-22

**Authors:** Alessandra Griffo, Carola Sparn, Fabio Lolicato, Friederike Nolle, Navid Khangholi, Ralf Seemann, Jean-Baptiste Fleury, Martin Brinkmann, Walter Nickel, Hendrik Hähl

**Affiliations:** a Department of Experimental Physics, Saarland University Saarbrücken Germany h.haehl@physik.uni-saarland.de; b Center for Biophysics, Experimental Physics, Saarland University Saarbrücken Germany; c Biophysical Engineering Group, Max Planck Institute for Medical Research Heidelberg Germany Alessandra.griffo@mr.mpg.de; d Heidelberg University Biochemistry Center Heidelberg Germany

## Abstract

The creation of free-standing lipid membranes has been so far of remarkable interest to investigate processes occurring in the cell membrane since its unsupported part enables studies in which it is important to maintain cell-like physicochemical properties of the lipid bilayer, that nonetheless depend on its molecular composition. In this study, we prepare pore-spanning membranes that mimic the composition of plasma membranes and perform force spectroscopy indentation measurements to unravel mechanistic insights depending on lipid composition. We show that this approach is highly effective for studying the mechanical properties of such membranes. Furthermore, we identify a direct influence of cholesterol and sphingomyelin on the elasticity of the bilayer and adhesion between the two leaflets. Eventually, we explore the possibilities of imaging in the unsupported membrane regions. For this purpose, we investigate the adsorption and movement of a peripheral protein, the fibroblast growth factor 2, on the complex membrane.

## Abstract 2

In this study, we prepare pore-spanning membranes that mimic the composition of plasma membranes and perform force spectroscopy indentation measurements to unravel mechanistic insights depending on lipid composition.

## Graphical abstract

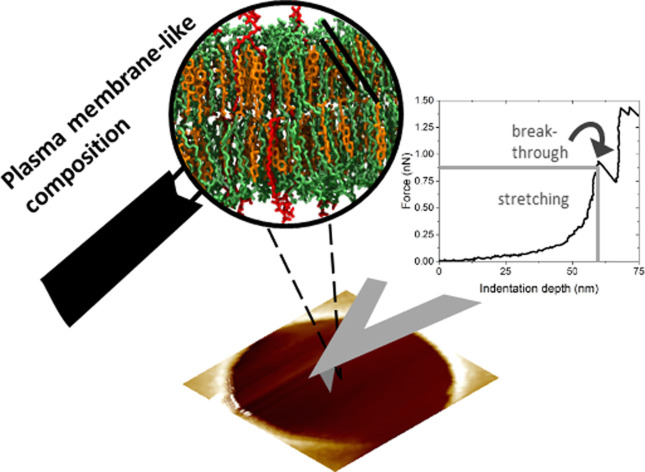

## Introduction

Lipid membranes form the cell membrane of almost all living organisms, such as eukaryotic cells, some prokaryotes, and several viruses. The membrane's ability to separate intracellular and extracellular ionic media makes it a functional capacitor that confines cells and organelles and regulates multiple translocation mechanisms.^1^ Together with the capacitive function, a set of physical properties of lipid membranes, including elasticity, tension, and curvature,^2^ strongly affects many of the processes that occur across the membrane. Examples are endocytosis, exocytosis, or conformational reshaping of proteins embedded in membranes.^3^ Also phenomena occurring at the protein–lipid interface as protein binding,^4^ oligomerization and insertion depend on the physical properties of lipid membranes and on their dynamic nature, which comprises processes, as *e.g.*, curvature fluctuations and diffusion. Supported lipid bilayers (SLBs) are a versatile and widely used system that enables access to a wide range of important membrane information due to their stability.^5^ The presence of the supporting substrate sacrifices, however, their dynamic nature and thus alters the reliability of observed processes occurring in and across the membrane.^6,7^ Liposomes, as an alternative system to SLBs, allow to explore bilayer properties without disturbing these dynamic processes, yet suffer from limited experimental access.^8^

**Fig. 1:**
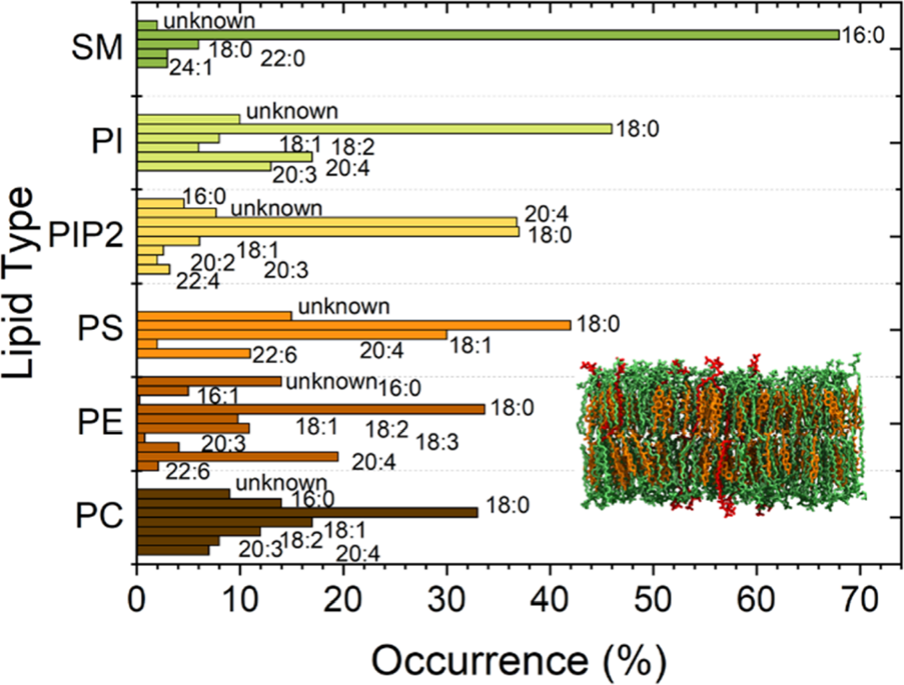
Fatty acid distribution of each of the natural lipids employed in the current study. The bars marked with *X* : *Y* show for a certain lipid type the fraction with chain length (number of C atoms) of the fatty acids *X* and number of unsaturated bonds *Y*. Lipid types are sphingomyelin (SM), phosphatidylinositol (PI), phosphatidylinositol bisphosphate (PIP2), phosphatidylserine (PS), phosphatidylethanolamine (PE), and phosphatidylcholine (PC). Data are adapted from Avanti Polar.

Moreover, membrane properties and functions depend also on chemical composition and polydispersity of the lipids present in the cell membrane.^9–12^ Biomimetic membranes should therefore be heterogeneous and polydisperse in lipid composition to resemble the mimicked living systems^2^ as much as possible. Each lipid contributes to complex regulation mechanisms across the cell. Charged lipids, such as phosphatidylserine (PS) and Phosphatidylinositol 4,5-Bisphosphate PI(4,5)P_2_, can regulate the binding affinity of proteins by electrostatic interactions.^13,14^ Sterols and sphingolipids are in particular known to influence the arrangement of lipids^12,15^ affecting both mechanical and chemical properties of lipid membranes and the resulting interactions with the surrounding environment. Also, cholesterol (CL) has been shown to significantly affect the bilayer stiffness. In addition, CL laterally segregates in domains in the outer leaflet of the membrane together with sphingomyelin, causing a considerable impact on the mechanics of the bilayer. Sphingomyelin (SM) has, due to its features, such as a high degree of saturation, asymmetric molecular structure, and extensive hydrogen-bonding properties, a very important role as a structural parameter in biological membranes. Furthermore, due to its tendency to interact with sterols, sphingomyelin largely regulates the cholesterol distribution within cellular membranes. More importantly, the tendency to form strong hydrogen bonds between sphingosine backbones of adjacent SM molecules is associated with an increase in compactness affecting the bilayer rigidiy.^16,17^ In the current work, we prepared biomimetic membranes on substrates with about 1 μm diameter circular holes. The pore-spanning membrane parts are a system between liposomes and SLBs featuring a better nanoscale observation than liposomes and a higher similarity to the natural situation than SLBs due to the absence of the solid support. These bilayer-model systems show water compartmentalization and bending rigidity similarly to liposomes,^18^ but are still accessible to atomic force microscopy (AFM) investigation, allowing the study of a wider range of phenomena, *e.g.*, monitoring the kinetics of protein activity on top of membranes.^9,19,20^ In order to design a system close to the cell membrane situation, we used a complex plasma membrane-like composition (PMC) of PC : PE : PS : PI : CL : SM : PI(4,5)P_2_ with ratios 33 : 10 : 5:5 : 30 : 15 : 2 for this study. Additionally, lipids from natural origin, which exhibit a higher distribution of the fatty acid chain lengths than the otherwise mostly employed synthetic lipids, were chosen for our biomimetic membranes (Fig. 1).

We investigated the effect of cholesterol and sphingomyelin on the membrane mechanics with the methods described below. In detail, we determined bilayer pre-stress and stretching elasticity for these heterogeneous membranes *via* AFM indentation and by analysis of optical micrographs of membrane standing in a microfluidic channel, while tuning the cholesterol and sphingomyelin content.

## Experimental

### Preparation of giant unilamellar vesicles

**Fig. 2:**
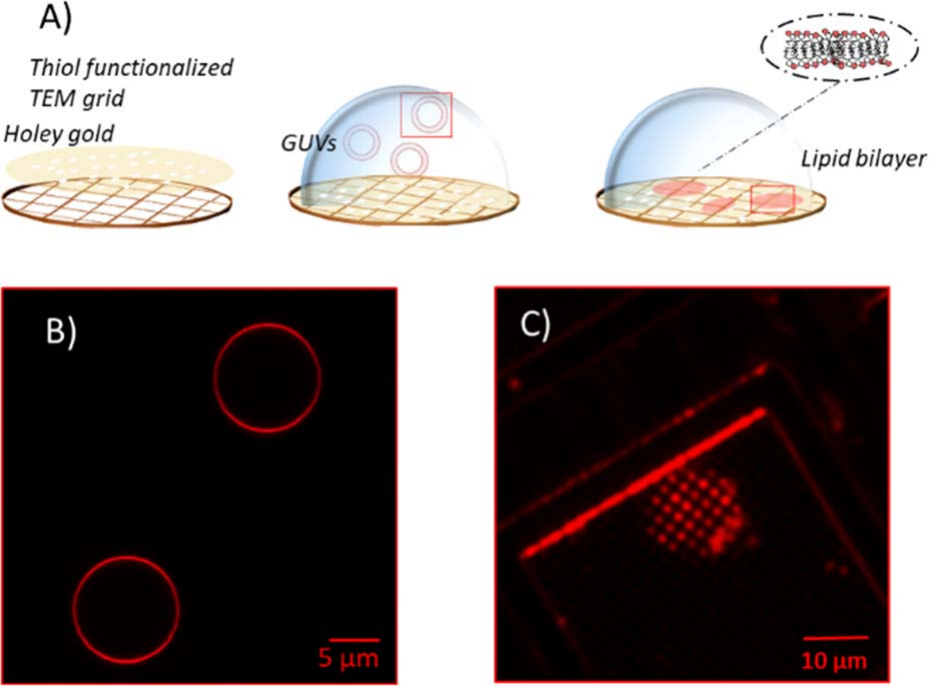
(A) Sketch of the process of GUVs bursting on top of hydrophilic functionalized TEM grids and corresponding fluorescence microscope images of GUVs (B) before and (C) after bursting on TEM grid. Lipids are stained with Rhod-PE.

Unsupported membranes have been produced by bursting giant unilamellar vesicles (GUVs) on top of mercapto-1-undecanol functionalized gold TEM grids (Plano GmbH, pore diameter of 0.8 and 1.2 μm), by adapting the methods described in the literature (Fig. 2A).^21–24^ Gold-coated TEM grids were functionalized with thiol self-assembled monolayers (SAMs). For that, they were first cleaned and made more hydrophilic *via* UV-Ozone treatment and then left overnight in a 1 mM solution of mercapto-1-undecanol in ethanol. After that, they were rinsed with ethanol to remove the unbound molecules and let dry. The functionalization was tested by water contact angle (WCA) measurements.

For creating the GUVs, a plasma membrane-like lipid composition (PMC) consisting of 30 mol% cholesterol (CL), 15 mol% sphingomyelin (SM), 33 mol% phosphatidylcholine (PC), 10 mol% phosphatidylethanolamine (PE), 5 mol% phosphatidylserine (PS), 5 mol% phosphatidylinositol (PI) and 2 mol% PI(4,5)P_2_ (Avanti Polar Lipids, Alabaster, AL) and a PMC lacking of CL and SF (PMC-) were prepared in chloroform with 1.5 mM concentration. All membrane lipids were purchased from Avanti Polar Lipids. They have been purified from natural extracts [bovine liver (PC, PE, PI), egg chicken (SM), porcine brain (PS, PI(4,5)P_2_), and ovine wool (CL)] and are therefore not uniform in alkyl chain length and unsaturated bond content (see Fig. 1). The GUVs were generated by electroformation, according to the protocol of Steringer *et al.*^22^ In detail, 5 μL of the chloroform solution containing the lipids was cast on platinum (Pt) wires, dried and immersed in sucrose solution (0.3 M). An AC-voltage of 1.5 V and a frequency of 10 Hz were applied for 40 min. After that, the frequency was reduced to 2 Hz for 20 minutes to allow the detachment of the GUVs from the Pt wires. The vesicles produced in this way (Fig. 2B) were collected, stored at 4 °C, and used no longer than two days after production.

Unsupported membranes were formed by diluting the stored GUVs suspension 1 : 50 in HEPES buffer (20 mM, supplemented with 300 mM NaCl, 25 mM MgCl_2_) and letting them burst, due to the difference in osmolarity between the sucrose inside the vesicle and the outer buffer, on the top of hydrophilic TEM grids mounted on a Petri dish. Bursting of GUVs was checked by fluorescence microscopy in the presence of Rhod-PE, as depicted in Fig. 2. Eventually, for the AFM imaging and indentation experiments the solution was exchanged with imaging buffer [HEPES buffer 20 mM, 100 mM NaCl, 1 mM Ethylenediaminetetraacetic acid (EDTA)].

### AFM imaging, force spectroscopy and data analysis

Atomic Force Microscopy (AFM) images and indentation experiments were acquired using a Dimension FastScanBio AFM (Bruker-Nano, Santa Barbara, CA, USA). MSNL-D probes (silicon nitride, spring constant 0.03 N m^−1^, resonance frequency 15 kHz, nominal tip radius 2 nm) were used for indentation experiments, while MSNL-D, SNL-D and PEAKFORCE-HIRS-F-B cantilevers (silicon nitride, spring constant 0.03 N m^−1^, 0.06 N m^−1^ and 0.12 N m^−1^ and resonance frequency 15 kHz, 18 kHz and 100 kHz, respectively, all probes purchased from Bruker AFM Probes, USA) were used for imaging. For each cantilever, the spring constant was calibrated by the thermal tune method. Peak Force Tapping® was used for imaging. Prior to indentation experiments, AFM imaging scans of the surface were performed, and the holes were checked for complete coverage. During imaging, scan sizes varied from 500 nm to 20 μm. In the force curve measurements, the approaching and retracting curves were recorded with zero surface delay and a ramp size of 100–200 nm. For each indentation experiment, hundreds of force distance curves, (*F*–*δ*), were recorded at the center of holes uniformly covered by the membrane. All experiments were performed at room temperature (controlled at *ca.* 20 °C).

The data obtained from AFM indentation experiments were processed using custom-made MATLAB scripts for baseline correction and contact point determination, and the breakthrough force of the first rupture event was extracted. Pre-stress and elasticity of the membrane were determined by fitting a model to the data that was derived by Jin *et al.*^25^ The model is an analytically exact solution of the Föppl-von Karman approximation for the ‘circular clamped elastic membrane under central point load with pre-stress’. It can thus describe situations with any pre-stress value within the two limiting cases with negligible pre-stress, which is characterized by a cubic dependency of the indentation force *F* on the indentation depth *δ*, and very large pre-stress, where the force is a linear function of the indentation depth. It is, however, not possible to write the general (*F*–*δ*) dependency in explicit form. Fitting the model to the data was therefore done using a custom-made MATLAB script (see ESI for details†).

### Free-standing membranes in microfluidic channel

**Fig. 3:**
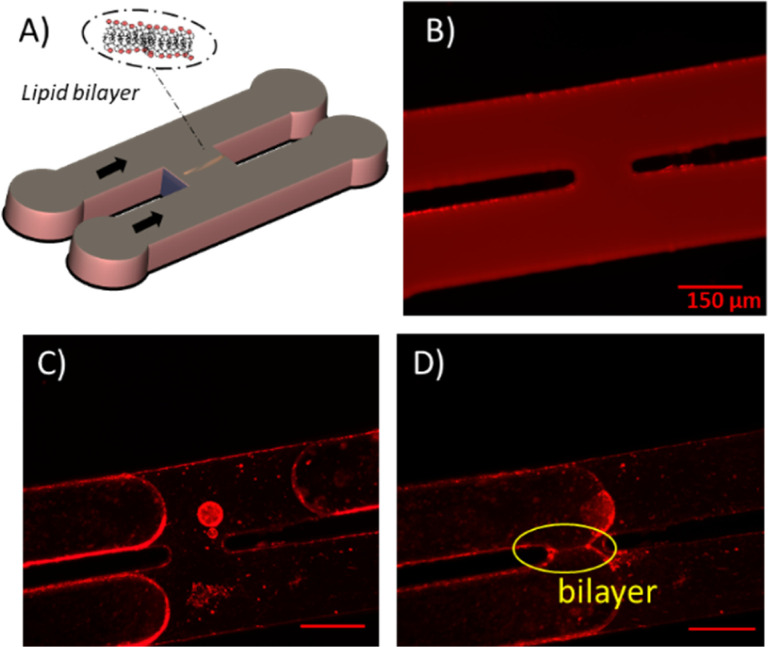
(A) Sketch of the design of the microfluidic setup featuring a H-junction. (B–D) Fluorescence microscope images (lipids are stained with Rhod-PE) monitoring the process of lipid bilayer formation from (B) initial flooding of the device with lipid-squalene solution, (C) slow injection of the buffer phase and (D) contacting the oil–buffer interfaces coated now with a lipid monolayer forming a bilayer at the contact.

As microfluidic channel setup, we employed an H-junction geometry previously tested as a platform to produce stable membranes to form unsupported lipid membranes in microfluidics,^26,27^*cf.*Fig. 3A and B. To guarantee a good wettability for the continuous oily phase at the channel walls, the microfluidic devices are fabricated from PDMS. The silicon elastomer base and curing agent (PDMS, Sylgard 184, Dow Corning, USA) are mixed in a ratio 10 : 1 and are casted on a silicon wafer, which exhibits structures produced by photolithography with a SU-8 photoresist, and cured for 6 h at 65 °C. After curing the PDMS elastomer, it is peeled off from the mold and exposed to nitrogen plasma (Diener electronic GmbH, Germany) together with an also PDMS-coated glass slide. The treated PDMS device is sealed with the glass slide and heated at 125 °C for 1–2 h to restore the hydrophobic properties of the PDMS. At the start of the experiment, this microfluidic device is filled with a continuous phase of squalene containing the lipids (5 mg mL^−1^). Subsequently, aqueous imaging buffer (Section 2.2) solution is injected into both channels of the device. Due to the wettability conditions, a thin squalene film remains between the buffer and the device walls, and the lipids contained in the squalene solution will decorate the oil–buffer interface forming a monolayer. A slow flow is created by hydrostatic pressure such that the lipid decorated oil–buffer interfaces are pushed through both channels and are brought in close vicinity at the junction. When the two monolayers touch each other, a zipping phenomenon driven by the intermolecular forces between the hydrophobic tails will occur, forming a bilayer (Fig. 3A, C and D). Optical images of the bilayer formation occurring in the chip were acquired with fluorescence microscopy at 10× magnification. The angle *θ* between the two leaflets of the bilayer can be obtained by analyzing the optical micrographs.

### Interfacial tension *γ* and bilayer tension *Γ*

Interfacial tension *γ* of a water–oil interface decorated with a monolayer of the lipid mixture is measured *via* the standard pendant drop method using a WCA goniometer (OCA 20, DataPhysics Instruments GmbH, Filderstadt, Germany). Briefly, a 1 mg mL^−1^ solution of lipid in squalene (0.858 g cm^−3^) is transferred in an optical glass cuvette. A drop of imaging buffer solution (0.997 g cm^−3^) is introduced from a needle that is dipped into the solution and the change of drop shape was observed over time, *i.e.* as a monolayer is formed at the interface. The droplet contour was fitted according to the Young–Laplace equation to obtain the interfacial tension *γ*. The decrease of *γ* due to the adsorption of lipids to the newly created interface is recorded over 20–30 minutes until a plateau is reached, denoting the interfacial tension of the monolayer decorated interface.

From the values of the interfacial tension *γ* and the bilayer contact angle *θ*, the bilayer tension *Γ* can be calculated using Young's equation:^28,29^

1 *Γ* = 2*γ* cos *θ*

Although the bilayer tension *Γ* and the pre-stress in the spanned bilayer *σ* are, in principle, the same quantity, namely the in-plane stress in the bilayer, they are named differently here to clarify their different origin: as the preparation conditions are different, it is not expected to measure the same in-plane stress in both experimental setups. Particularly, the bilayer tension *Γ* in the microfluidic setup results from the interfacial tension *γ* and the adhesion energy Δ*W* between the two bilayer leaflets as

2 Δ*W* = 2*γ* − *Γ* = 2*γ*(1 − cos *θ*).

## Results

**Fig. 4:**
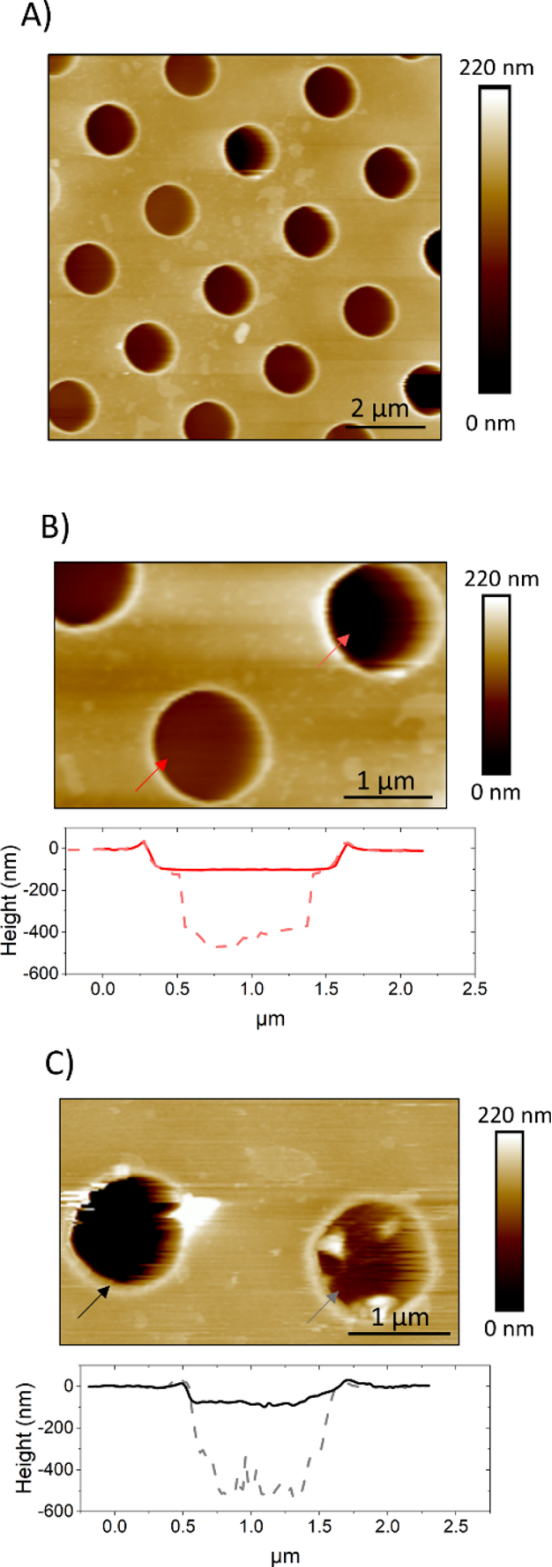
AFM images showing PMC (A and B) and PMC^−^ (C) lipid membranes spanning holes in a functionalized Au-grid. Section analysis in B and C show intact (red and black) and broken (light red and gray dashes) membranes respectively for PMC and PMC^−^. Images were recorded at a PeakForce Setpoint of 280 pN. Additional images of PMC and PMC^−^ are reported in Fig. SI1.†

Unsupported lipid membranes with PM-like composition (PMC) or with a PM-like composition without CL and SM molecules (PMC^−^) spanning hydrophilic coated TEM grids (pore diameter 1.2 μm and 0.8 μm) were formed. AFM scans (Fig. 4) revealed the presence of membranes over the selected spots. The AFM cross sections (Fig. 4b and c) show that the membrane covers the rim around the hole, which has a height of *ca.* 20 nm, and is recessed inside the hole below the actual substrate's surface to a depth of *ca.* 70–100 nm.

**Fig. 5:**
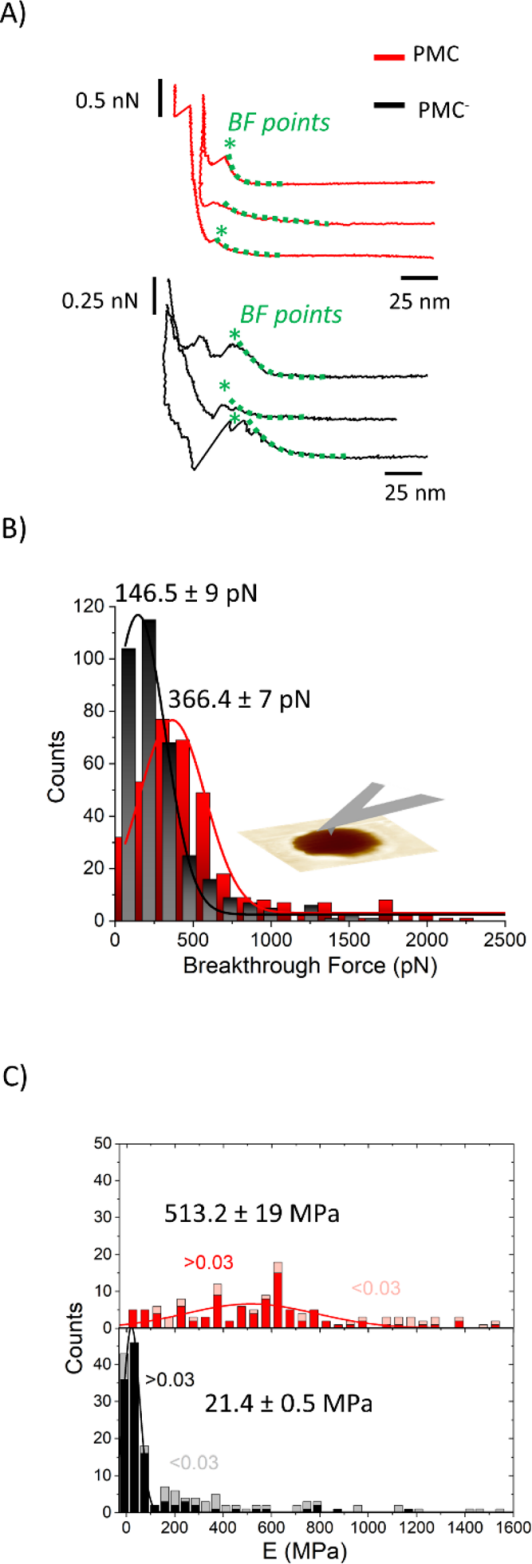
(A) *F*–*δ* curves on PMC (red) and PMC^−^ (black) membranes. (B) Histograms of the breakthrough force values (BF) for PMC (red) and PMC^−^ composition (black) fitted using a Gaussian function. (C) Stacked histograms reporting the Young's modulus *E* for PMC (red squares for *δ* > 0.03 and pink for *δ* < 0.03) and PMC^−^ (black squares for *δ* > 0.03 and gray for *δ* < 0.03). Additional *F*–*δ* curves are reported in Fig. SI2.†

We performed AFM force–indentation (*F*–*δ*) measurements in the center of membrane-covered holes (Fig. 5A). From these data, we extracted the three parameters of breakthrough force (BF), elastic modulus (*E*), and bilayer pre-stress (*σ*) in order to understand the effect of CL and SM on the mechanical properties of the PMC membranes. BF is defined as the maximum force the membrane can withstand before it ruptures, indicated by an abrupt decrease in force. It can be correlated to the packing density of lipids in the membrane.^30^ The *F*–*δ* curves reported in Fig. 5A show a nonlinear response in the region between the snap in/contact point, which is due to tip–surface interactions like capillary forces, and the BF point. This region was fitted (green dashed lines) with a model from non-linear elastic theory of membranes^25^ (see ESI†) enabling an estimation of both the elastic modulus *E* of the bilayer and its pre-stress *σ*.

The histograms shown in Fig. 5B are a collection of BFs from PMC and PMC^−^ membranes. They exhibit peaks that are fitted by Gaussian functions centered at (366 ± 7) pN for PMC and at (146 ± 9) pN for PMC^−^.

In Fig. 5C, *E* values obtained from the fitting procedure are plotted. Since no dependency on the hole radius was observed, we combined data from membranes spanning holes of 0.8 μm and 1.2 μm diameter. The maximum indentation depth reached in the experiment, however, affects the accuracy of the estimation of the Young's modulus. Since the stretching of the bilayer is more pronounced for larger indentations, the elastic modulus will be more important for the force response of the bilayer and thus, the estimation of *E* is improved. From a plot of the obtained *E*-values *vs.* the scaled maximum indentation depth on hole radius *δ*/*R* we decided for a threshold of *δ*/*R* = 0.03. Only values above this threshold were used for the estimation of the mean value of *E*. In Fig. 5C, histograms of the *E*-value distributions are presented where the excluded values with *δ*/*R* < 0.03 are displayed as lighter shaded bars showing that they are mainly high value outliers in the distributions. Values above this threshold (red and black bars of Fig. 5C) are fitted with a Gaussian distribution with a peak value for the elasticity modulus *E* at (513 ± 19) MPa and (21.4 ± 0.5) MPa for the PMC (red squares) and PMC^−^ (black squares) compositions, respectively.

**Fig. 6:**
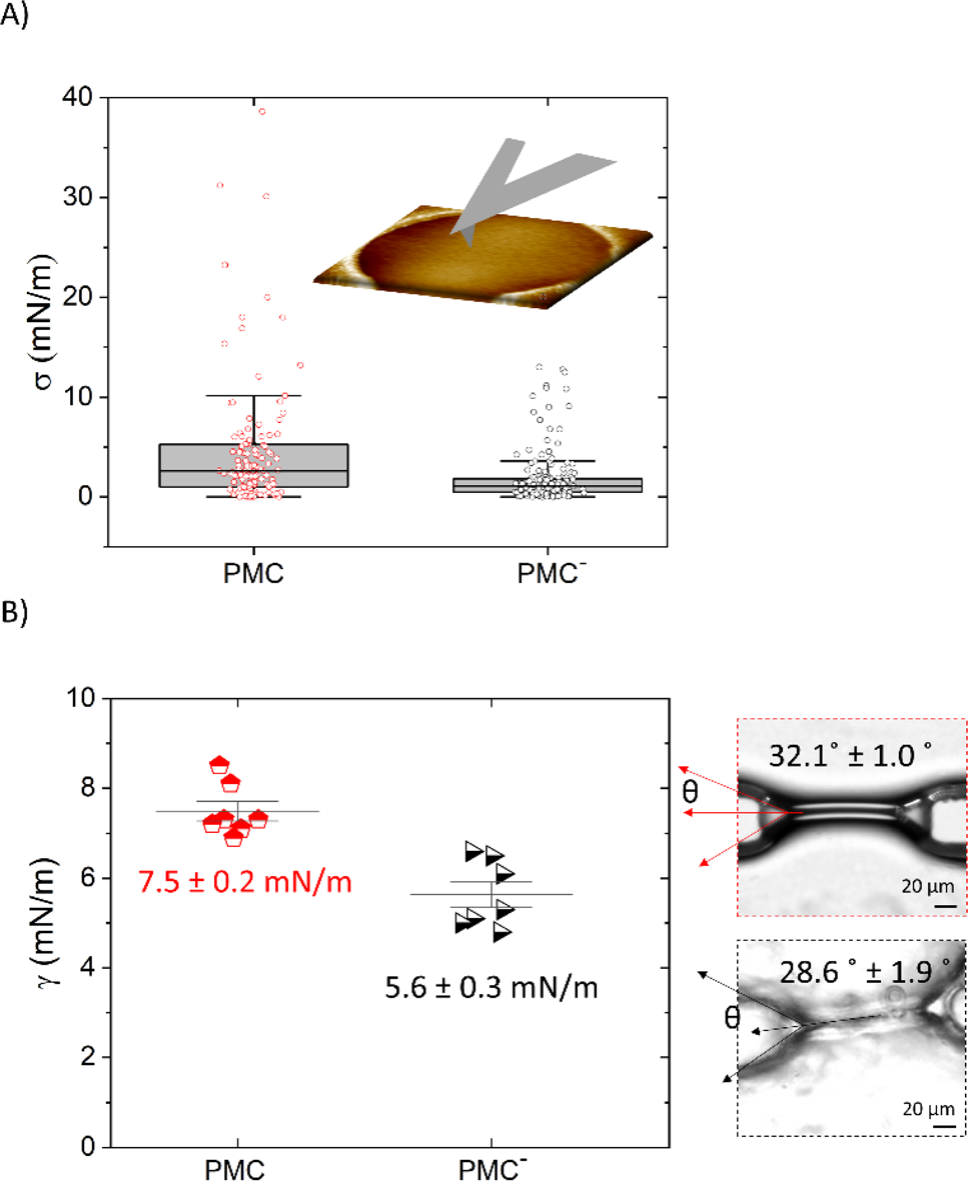
(A) Box plots of pre-stress *σ* (±SE) values recorded *via* AFM indentation measurements for PMC and PMC^−^ compositions. (B) Plots of interfacial tensions recorded *via* pendant drop measurements and optical micrographs of the membranes formed in a microfluidic chip showing respectively the interfacial tension *γ* and the bilayer angle *θ* for PMC and PMC^−^ compositions. *θ* and *γ* are used to calculate the bilayer tension *Γ* with the Young–Laplace equation.

Pre-stress values *σ* extracted from the *F*–*δ* measurements that are shown in Fig. 5A are reported in Fig. 6A. We find that the value for *σ* is reduced from (5.2 ± 0.8) mN m^−1^ for the PMC membrane to (2.0 ± 0.2) mN m^−1^ for the PMC^−^ membrane. For qualitative comparison, the tension in the bilayer for both compositions was also determined from the contact angle *θ* of two contacted lipid monolayers in a microfluidic setup. Together with the interfacial tension *γ* of the monolayer, which was measured using the drop shape analysis of a pendant drop, and employing the Young–Laplace equation (eqn (1)),^26^ bilayer tension values *Γ* of (13 ± 4) mN m^−1^ and (10 ± 1) mN m^−1^ (Fig. 6B) were obtained for PMC and PMC^−^ membranes, respectively. These values relate *via*eqn (2) to adhesion energies Δ*W* between the sheets of (2.3 ± 0.2) mJ m^−2^ and (1.4 ± 0.3) mJ m^−2^.

In addition to the mechanistic insights, we used the imaging capabilities of the AFM to resolve the bilayer surface. Our choice to employ unsupported membranes for AFM investigation is guided by the need to construct platforms for protein observation and detection of its related processes, *i.e.*, diffusion, kinetics assembly, pore forming mechanisms. Fig. SI3† shows a series of AFM images of the free-standing part of the bilayer recorded while the protein fibroblast growth factor 2 (FGF2-GFP) was added to the solution above the bilayer, using a fluid cell setup. These AFM time-lapse frames show the adsorption of this peripheral protein across the bilayer. However, due to the fluidity of lipid membranes and the difficulty of imaging standing portions of lipid membranes, it is hard to picture a clear statement from the reported images.

## Discussion

The described characterization allowed us to gain insight to the mechanics of membranes. The BF values of (366 ± 7) pN and at (146 ± 9) pN recorded by AFM for both unsupported PMC and PMC^−^ membranes imply a difference in the compactness of the two membranes. The PMC membranes exhibit a higher breakthrough force, meaning that the membrane can resist the applied force more strongly. This observation matches quite well with the condensing effect of cholesterol,^31^ which is well known to regulate the membrane fluidity and produce an ordering of the lipids, leading to a decrease in its permeability.^31–33^ Such insights have been suggested already by studies of SLBs^34–36^ where information about the chemical composition of the membrane, its lipid packing, and the surrounding environment were gained from the BF. Sullan *et al.*^37^ found that an increase of the BF was observed in the SM/Chol-enriched liquid-ordered domains (Lo) compared with the DOPC-enriched fluid-disordered phase (Ld). While the BF values they reported for SLBs were in the nN-range, our results for free-standing membranes fall, however, in the pN-range. Such differences are reasonably explained by the absence of a solid support. In our setup, the AFM cantilever causes a tensile stress and thus a stretching of the whole free-standing part of the membrane. On the molecular scale, this translates into a reduction of the packing density, which facilitates rupture of the bilayer (at the BF point) through the increase in inter-molecular distances. In the SLB, in contrast, the AFM tip causes a localized compressive stress in order to punch through the bilayer, inducing an even higher packing density.

A closer insight into the mechanics of PMC lipid membranes and into the interplay of cholesterol and sphingomyelin can be obtained by extracting the stretching elasticity of the bilayer from the *F*–*δ* curves. Therefore, a theoretical description derived from the Föppl–von Karman model of elastic membranes was used. For a clamped elastic membrane under a central point load, a linear relation between force and indentation is predicted if the indentation is small and the pre-stress is not negligible. For higher indentations and small or negligible pre-stress, stretching of the membrane dominates and leads to a cubic relation depending on the elasticity for the membrane,^38^*F* ∝ *Eδ*^3^. As an approximation, a linear superposition of those limiting behavior descriptions is used in similar studies^39–41^3
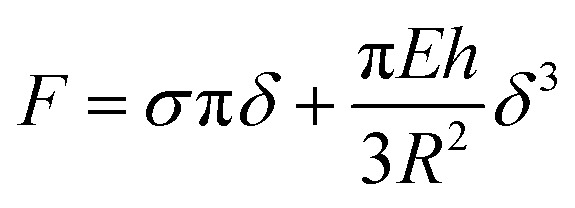
where *h* is the bilayer thickness, here assumed as 6.7 nm for PMC membranes and as 6.0 nm for PMC^−^ membranes,^42,43^*R* the hole radius, *E* the elastic modulus and *σ* the pre-strain of the membrane. Note, however, that in this setup the membrane is stretched laterally and thus only its lateral 2D elasticity *E*^2D^ is probed. *E*^2D^ is related to *E* as *E*^2D^ = *E* × *h* under the assumption of an isotropic material, which is not strictly valid in this case. We nevertheless state the values for *E* for a better comparability. In the data of this study (Fig. SI4 and SI5†), both behaviors, linear and cubic, can be observed: a constant slope for low indentations and a deviation from the linear dependency for higher indentations of *δ*/*R* ≳ 0.03. Therefore, the accuracy with which *E* is determined increases with higher *δ*. Physically, this reflects the notion that the more the membrane is indented, the more it is stretched, and the more important Young's modulus becomes.

Since the data of this study fall, however, mostly in the transition region between linear and cubic behavior, where the inaccuracy of the simple model is highest, we chose a slightly more complicated, yet analytically exact solution of the theoretical model by Jin *et al.*^25,44^ (see ESI for details†). Data shown in Fig. 5C and 6A were obtained by fitting this model to the experimental *F*–*δ* curves. Compared to the approximation of eqn (1), we obtained slightly lower values for *E* and *σ* as well as a lower scattering (a comparison between the approximate and exact solution of the model is reported in Fig. SI4†). The obtained elasticity values recorded upon membrane indentation vary from 10 to 1000 MPa. Thereby, lower elasticity values are found for higher maximum indentation depths, with the smallest near 100 and 30 MPa for PMC and PMC^−^ membranes, respectively (Fig. SI4†). The latter values correspond to the maximum achievable membrane stretching.

The obtained results on membrane mechanics match with many works reported in literature^15,45^ where the role of SM and CL is attributed to an increase in stiffness. Yet, in most of the studies elasticity of membranes has been extensively probed for supported lipid bilayers and vesicles, but rarely for unsupported membranes. To enable a better comparison for the data determined from this study with elasticity measurements from vesicle indentation,^46,47^ where normally the bending rigidity is probed, we calculated this property from our measurements *via*4
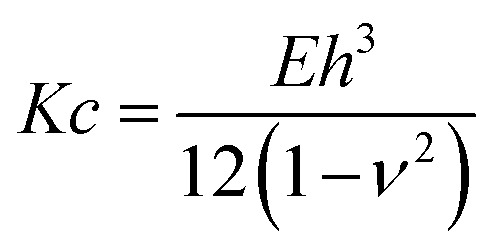
where *ν* is the Poisson ratio kept as 0.485,^48^*E* the elastic modulus and *h* the membrane thickness. We find values of about 200 × 10^−19^ J and 9 × 10^−19^ J at maximum indentation depths for PMC and PMC^−^ membranes, respectively. Such values are slightly above those typically reported in the literature for membranes not containing rigid molecules such as SM and CL^9,20,49^ and could be attributed to the presence of rigid-like lipids such as SM and CL that increase the membrane rigidity. Our results report a remarkable membrane rigidity, which is even larger compared to cholesterol enriched membranes.^15,50^ Sphingomyelin, in fact, increases the packing density of the membrane due to its hydrogen bond forming capacity with neighboring lipids.^17^ Also, the PI(4,5)P_2_ may cause, due to its conical shape, a stiffening of the membrane^15^ and an increase of the lateral pressure in the acyl chain region.^51^

Studies on tension and pre-stress instead have been carried out more extensively also for monodisperse and unsaturated lipids on unsupported membranes.^21,23,52^ In the work of Kocun *et al.*,^21^ site specific force indentation experiments on porous substrates were performed to determine membrane tension as a function of lipid composition. Although they used a similar setup, their pure POPC or DOPC membranes showed only a linear force–indentation behavior, which did not allow for a determination of the bilayer's elasticity. They obtained, however, an apparent spring constant, which depends on the preparation-imposed pre-tension of the bilayer and is comparable to (*σ*π) in our analysis. They observed an increase in the apparent spring constant for DOPC membranes from about 1.0 mN m^−1^ to 3.5 mN m^−1^ after the addition of cholesterol, which matches well with our findings, where the addition of the two rigid-like components cholesterol and sphingomyelin led to a shift from 2 mN m^−1^ to about 5 mN m^−1^. In the case of the spanned membrane, the tension of the membrane is influenced by the adhesion to the substrate surrounding the hole, where a higher adhesion leads to a higher tension.^52,53^ For the membranes in the microfluidic setup, the tension is directly given by the adhesion between the sheets *via*eqn (2). For both situations, a higher tension thus means an increase in adhesion; in one case, to the substrate and, in the other case, between the leaflets. It is interesting to note that the addition of CL and SM in the membrane leads, in both cases, to an increased adhesion. However, the ratio of this increase seems too large to be attributed solely to an increased molecular density in the bilayer.

Our observations shed light on the function of sterols and sphingolipids on more complex compositions, namely on membranes composed by seven different lipid types, each of them featuring polydispersity in unsaturation degree and carbon chain length.^45^ Previous studies showed that for certain proteins, *e.g.* the FGF2 used in our experiments, both binding and translocation are highly enhanced when cholesterol and sphingomyelin are present.^54^ The underlying mechanism remains not entirely elucidated, but it's probable that variations in tension and the lipids' inherent properties play significant roles in this phenomenon.

## Conclusions

The technology of membrane biomechanics is continuously in evolution and currently many systems are studied, from *in vitro* constructed liposomes,^55^ pore spanning membranes,^35^ synthetic cells^56^ to entire cells,^57^ using a variety of techniques, including AFM, fluctuation spectroscopy and micropipette aspiration. In our study, we employed atomic force microscopy, atomic force indentation and less extensively also microfluidics, to achieve insights into mechanical properties, such as bilayer tension and elasticity, morphological features, and protein-membrane interaction studies on unsupported membranes. Starting from the pore spanning technology we used biomimetic lipid membranes for which we chose a plasma membrane-like composition from natural lipids, to shed light on the properties of complex membranes, which feature lipid heterogeneity and polydispersity. The focus was mainly on the role of cholesterol and sphingomyelin, for which there is still a lack of information. The shift in the values of elastic modulus, of the breakthrough force as well as in the adhesion energies between PMC and PMC^−^ shows how the combination of different bilayer components affects the compactness and the mechanics of the membranes. This led us to the conclusion that cholesterol and sphingomyelin increase membrane tension and rigidity even in more complex PMC compositions. Eventually, we propose the described unsupported membranes as an alternative to supported ones as model for systematic studies with more natural dynamics.

## Data availability

The source code for the MATLAB functions utilized for fitting the data in this study is available on Zenodo, under the DOI: https://10.5281/zenodo.10854432.

## Author contributions

A. G. performed the experiments and designed the work, F. N., N. K., J.-B. F. helped for the microfluidic experiments, C. S., F. L. helped in vesicle productions and W. N. supported the scientific discussion and in planning the project, M. B. helped in choosing the mathematical model, H. H. helped in the data analysis and designed the work. All the authors contributed to the manuscript writing. A. G. and H. H are shared corresponding authors.

## Conflicts of interest

The authors declare no competing financial interest.

## Supplementary Material

RA-014-D4RA00738G-s001
